# Exploring the Association between Citrus Nutraceutical Eriocitrin and Metformin for Improving Pre-Diabetes in a Dynamic Microbiome Model

**DOI:** 10.3390/ph16050650

**Published:** 2023-04-26

**Authors:** Thais Cesar, Mateus Kawata Salgaço, Victoria Mesa, Adilson Sartoratto, Katia Sivieri

**Affiliations:** 1 Graduate Program in Food, Nutrition and Food Engineering, Campus Araraquara, São Paulo State University (UNESP), Araraquara 14800-060, SP, Brazil; thais.cesar@unesp.br (T.C.);; 2 INSERM, UMR-S 1139 (3PHM), Faculty of Pharmacy, Université Paris Cité, F-75006 Paris, France; 3 Food and Human Nutrition Research Group, School of Nutrition and Dietetics, Universidad de Antioquia (UdeA), Medellín 050010, Antioquia, Colombia; 4 CPQBA-UNICAMP, Paulínia 13148-218, SP, Brazil

**Keywords:** lemon flavonoids, microbiota, prebiotic, short-chain fatty acids

## Abstract

Pre-diabetes is recognized as an altered metabolic state, which precedes type 2 diabetes, and it is associated with great dysfunction of the intestinal microbiota, known as dysbiosis. Natural compounds, capable of reducing blood glucose without side effects and with a beneficial effect on the microbiota, have been studied as substitutes or adjuvants to conventional hypoglycemic agents, such as metformin. In this work, the effect of the nutraceutical Eriomin^®^, a mixture of citrus flavonoids (eriocitrin, hesperidin, naringin, and didymin), which reduces glycemia and increases glucagon-like peptide-1 (GLP-1) in pre-diabetic patients, was tested in the Simulator of Human Intestinal Microbial Ecosystem (SHIME^®^), inoculated with pre-diabetic microbiota. After treatment with Eriomin^®^ plus metformin, a significant increase in acetate and butyrate production was observed. Furthermore, sequencing of the 16S rRNA gene of the microorganisms showed that Eriomin^®^ plus metformin stimulated the growth of *Bacteroides* and *Subdoligranulum* genera. *Bacteroides* are the largest fraction of the intestinal microbiota and are potential colonizers of the colon, with some species producing acetic and propionic fatty acids. In addition, *Subdoligranulum* species are associated with better host glycemic metabolism. In conclusion, Eriomin^®^ associated with metformin improved the composition and metabolism of the intestinal microbiota, suggesting a potential use in pre-diabetes therapy.

## 1. Introduction

The onset of pre-diabetes is characterized by biochemical and metabolic impairment, leading to insulin resistance and increased fasting blood glucose (≥5.6 to 6.9 mmol/L). In addition, pre-diabetes precedes the onset of type 2 diabetes (T2D) by a few months to years, a crucial period for modifying and reversing to normality. A recommended clinical strategy for pre-diabetes is treatment with hypoglycemic agents, such as metformin, combined with lifestyle changes [1]. The continuous use of metformin at an adequate dose delays the development to T2D, improves insulin sensitivity, and reduces fasting blood glucose [2]. Despite the currently recognized benefits of metformin in diabetes, and also in reducing the risk of some types of cancer and in the effectiveness of cancer treatment in diabetic patients [3], it has some limitations in susceptible individuals due to its side effects and partial reversal in reducing blood glucose [4].

Alternatively, natural compounds such as flavonoids, capable of reducing blood glucose without side effects, have been studied as substitutes or adjuvants for conventional hypoglycemic therapy. The nutraceutical Eriomin^®^, produced from lemon flavonoids (eriocitrin, hesperidin, naringin, and didymin), has previously been shown to lower blood glucose, glycated hemoglobin (HbA1c), and oral glucose tolerance test (OGTT) results, as well as significantly increase GLP-1 in pre-diabetic patients with no drugs or dietary supplements [5,6]. As per other polyphenol compounds, lemon flavonoids are deglycosylated before being absorbed in the upper intestine, but most of them are degraded by intestinal bacteria in the colon, leading to enhancement of their bioavailability. Furthermore, flavonoids can modulate the intestinal microbial population structure, which might be associated with their bioactivities [7].

Recent evidence has shown that intestinal microbiota is a primary factor in the development of pre-diabetes and T2D, in addition to genetic and environmental factors [8]. Studies with diabetic animals and humans have shown dysbiotic microbiota, with less diversity and abundance of beneficial bacterial genus, such as *Bifidobacterium*, *Bacteroides*, *Faecalibacterium*, *Akkermansia*, and *Roseburia* [9]. On the contrary, drug therapy with metformin has been associated with a reduction in *Clostridium* spp., but with a greater abundance of beneficial genera, such as *Arkkermansia*, *Bifidobacterium*, and *Lactobacillus* [8]. Metagenomic analysis has further shown that metformin increases short-chain fat acid production (mainly propionate and butyrate) and bile acid conversion, improves intestinal permeability by reducing endotoxins, and increases the production of GLP-1 and gut hormone peptide YY (PYY) [10]. These data suggest that the effect of metformin on hyperglycemia could be indirect via intestinal microbiota, although more clinical studies are needed for definitive and direct evidence.

Based on these data, the hypothesis of this study was that Eriomin^®^ combined with metformin may increase the effectiveness of hypoglycemic therapy through effects on intestinal microbiota. Therefore, Simulator of Human Intestinal Microbial Ecosystem (SHIME^®^), inoculated with fecal microbiota of pre-diabetic volunteers, was used to study the combined effect of Eriomin^®^ plus metformin. It is expected that the responses obtained in this preclinical study, using citrus flavonoids as nutraceuticals together with conventional drug therapy, can point to new perspectives in the prevention and treatment of pre-diabetes.

## 2. Results

### 2.1. Effect of Eriomin^®^ Combined with Metformin on Short-Chain Fatty Acid (SCFAs) Production by the Gut Microbiome

**Figure 1:**
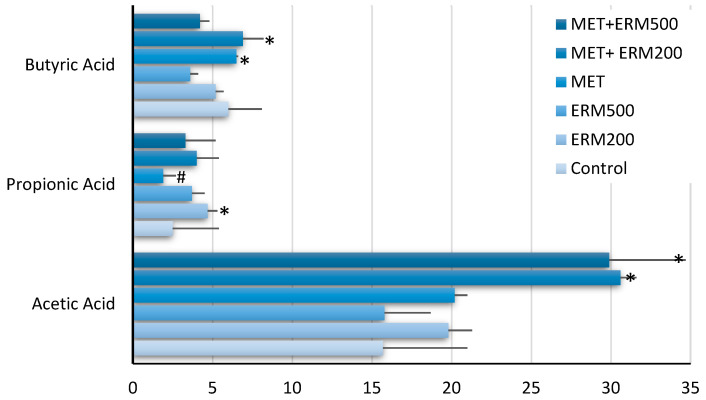
Eriomin^®^ associated with metformin-induced changes in the metabolic activity of the intestinal microbiome in vitro. The production of SCFAs was monitored for 7 days of fermentation with the following treatments: Control (microbiota without treatment); 200 mg/d Eriomin^®^ (ERM200); 500 mg/d of Eriomin^®^ (ERM500); 500 mg/d of metformin (MTF), 500 mg/d of metformin + 200 mg/d of Eriomin^®^ (MTF + ERM200); 500 mg/d of metformin + 500 mg/d of Eriomin^®^ (MTF + ERM500). Data are expressed as the average of each SCFA produced. The symbols * and # mean statistical increase or decrease, respectively, between treatments for each SCFA (*p* ≤ 0.05).

SCFAs production was determined to assess the potential of nutraceutical Eriomin^®^ associated with metformin in improving the intestinal microenvironment, as shown in Figure 1.

It was shown that 200 mg (ERM200) and 500 mg (ERM500) of Eriomin^®^ associated with 500 mg of metformin (Met) doubles the production of acetic acid compared to treatments alone or the control (*p* ≤ 0.001). In addition, propionic acid was increased by ERM200 and decreased by Met (*p* ≤ 0.04), while butyric acid was increased by MTF500 + ERM200 regarding the control (*p* ≤ 0.01).

### 2.2. Effects of Eriomin^®^ Combined with Metformin on the Gut Microbial Composition

**Figure 2:**
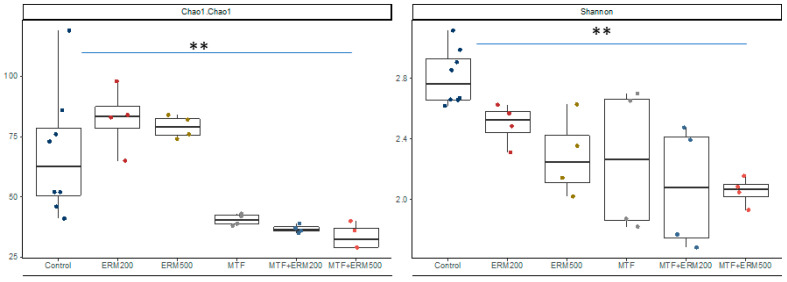
Variations in the alpha diversity of Eriomin^®^ combined with metformin. Legend: Control, microbiota without treatment; ERM200, 200 mg/d of Eriomin^®^; ERM500, 500 mg/d of Eriomin^®^; MTF, 500 mg/d of metformin; MTF + ERM200, 500 mg/d of metformin + 200 mg/d of Eriomin^®^; MTF + ERM500, 500 mg/d of metformin + 500 mg/d of Eriomin^®^. The symbol ** mean statistical increase or decrease, respectively, between treatments for alpha diversity index (*p* ≤ 0.05).

Alpha diversity, represented by Shannon’s index, and richness, estimated by Chao1, showed that the diversity was altered by treatment (*t*-test, Shannon *p* = 0.005, Chao1 value *p* = 0.0008) (Figure 2). Interestingly, no difference was detected for richness among ERM200 and ERM500 regarding the control (Chao 1), but all treatments, isolated or combined, reduced the diversity (Shannon index) of the microbiota when compared to the control.

**Figure 3:**
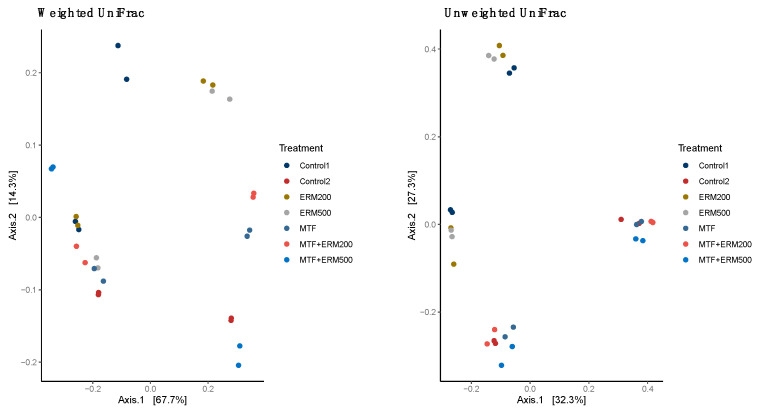
Principal coordinate analysis (PCoA) of the weighted and unweighted UniFrac distances variations in beta diversity of Eriomin^®^ combined with metformin. Legend: Control, microbiota without treatment; ERM200, 200 mg/d of Eriomin^®^; ERM500, 500 mg/d of Eriomin^®^; MTF, 500 mg/d of metformin; MTF + ERM200, 500 mg/d of metformin + 200 mg/d of Eriomin^®^; MTF + ERM500, 500 mg/d of metformin + 500 mg/d of Eriomin^®^.

In addition, the microbiota beta diversity, verified by principal coordinate analysis (PCoA) of weighted and unweighted UniFrac distance variations, revealed that ERM200 and ERM500 clustered with the control. Furthermore, the combination of both with metformin tended to cluster as well. Additionally, analysis of the permutational variation (PERMANOVA) for unweighted UniFrac distances showed a significant difference between treatments (*p* = 0.01) (Figure 3).

**Figure 4:**
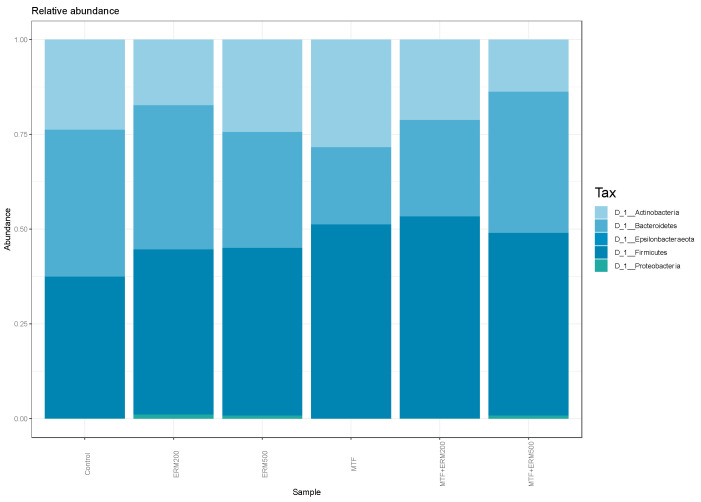
Histogram of the community composition of gut microbiota at the phylum level. Control, microbiota without treatment; ERM200, 200 mg/d of Eriomin^®^; ERM500, 500 mg/d of Eriomin^®^; MTF, 500 mg/d of metformin; MTF + ERM200, 500 mg/d of metformin + 200 mg/d of Eriomin^®^; MTF + ERM500, 500 mg/d of metformin + 500 mg/d of Eriomin^®^.

Analysis of the prevalent phyla in the microbiota of pre-diabetic patients, after treatment with Eriomin^®^ and metformin alone or in association, showed a predominance of *Firmicutes*, *Bacteroidetes,* and *Actinobacteria*, as shown in Figure 4.

**Figure 5:**
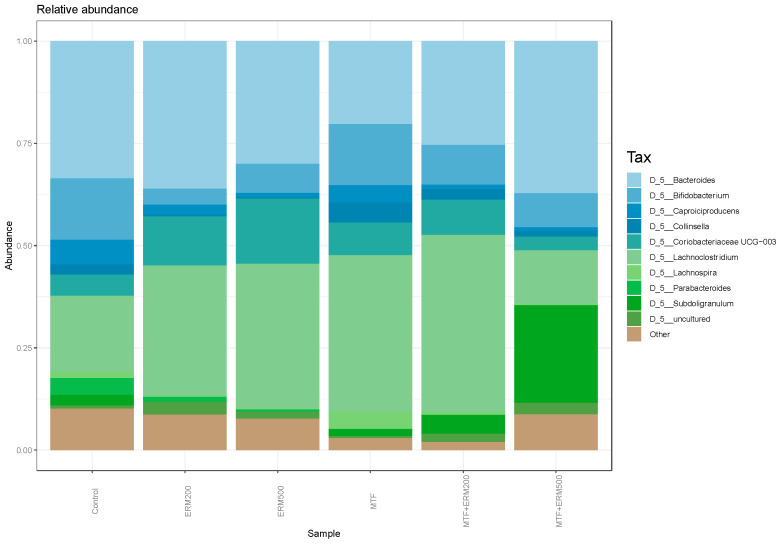
Histogram of the community composition of gut microbiota at the genus level. Legend: Control, microbiota without treatment; ERM200, 200 mg/d of Eriomin^®^; ERM500, 500 mg/d of Eriomin^®^; MTF, 500 mg/d of metformin; MTF + ERM200, 500 mg/d of metformin +200 mg/d of Eriomin^®^; MTF + ERM500, 500 mg/d of metformin +500 mg/d of Eriomin^®^.

To study the essential differences in community structure, genera with relative abundances less than 1% were excluded. The top 10 genera with higher relative abundances were selected for comparison, as shown in Figure 5.

The taxonomic assignment performed in ascending colon vessels of SHIME^®^ showed an increase in the *Lachnoclostridium* genus and *Coriobacteriaceae* family (*p* ≤ 0.0001) for all treatments except MTF + ERM500 when compared to the control. When treated with MET alone, an increase in *Bifidobacterium* and *Collinsella* genera was observed (*p* ≤ 0.0001). The combination of MTF + ERM500 showed an increase in *Bacteroides* and *Subdoligranulum* genera compared to the control (*p* ≤ 0.0001).

## 3. Discussion

Pre-diabetes is currently recognized as a relevant metabolic state that must be treated urgently in order to prevent the development of diabetes and associated diseases [11]. Among the available treatments, which include diet, physical activity, and weight loss, metformin is a first-line drug recommended to treat pre-diabetes and the onset of T2D [1]. The present study investigated, for the first time, the effects of the association of the nutraceutical Eriomin^®^ and metformin on the intestinal microbiota of individuals with pre-diabetes. A microbiome model, known as the Simulator of Human Intestinal Microbial Ecosystem (SHIME^®^), was loaded with human pre-diabetic microbiota to study the effects of metformin associated with a flavonoid prebiotic named Eriomin^®^. Our results revealed that treatment with Eriomin^®^ and metformin, alone or in combination, had a great influence on the metabolism and composition of the pre-diabetic microbiota.

It was found in this study that the combination of metformin plus 200 mg of Eriomin^®^ increased the short-chain fat acids production, such as acetate and butyrate, compared to the control, while metformin increased only butyrate. It is important to highlight that for the SHIME^®^ model, given the absence of absorption by the epithelium, these results showed a simulated production of SCFAs in the intestinal lumen. Recently, Meng et al. [12] showed that eriocitrin, the main flavonoid of Eriomin^®^, can increase the content of SCFAs in the colon of mice. SCFAs produced by intestinal microorganisms are considered potential metabolic compounds for preventing glucose metabolic disorders and insulin resistance in diabetic patients [13]. Increasing the concentration of acetate in the intestine improves the energy metabolism of the host, mobilizing the secretion of the intestinal hormone GLP-1 and the peptide YY. It also modifies appetite by reducing body lipolysis and systemic levels of pro-inflammatory cytokines and leads to increased energy expenditure and fat oxidation [14]. In addition, butyrate will maintain the stability and integrity of the intestinal barrier, decreasing intestinal permeability and circulating endotoxins, in addition to reducing inflammation and oxidative stress [15]. A previous study also revealed that metformin increases the rate of butyrate production in the gut microbiome [16]. Therefore, the passage of metformin through the intestine and its subsequent absorption are fundamental steps in the pharmacokinetics and pharmacodynamics of this drug. More recent evidence indicates that the intestine must be the main site responsible for the hypoglycemic effect of metformin [17].

Due to the influence of the intestinal microbiota on the absorption and metabolism of nutrients and non-nutritive compounds in the intestine, a fundamental role in the pathogenesis of pre-diabetes and diabetes has been assigned to these microorganisms. Therefore, the intestinal bacterial components and metabolites affect the progression of T2D by regulating immunity, inflammation, and metabolism [18].

It is known that microbiota affects the bioactivity of phenolic compounds, including flavonoids, promoting the release of metabolites that benefit the host [19]. In the current study, there was no change in the alpha diversity (Chao 1) of the microbiota upon treatment with Eriomin^®^ (200 or 500 mg). However, the beta diversity revealed a significative difference between Eriomin^®^ with metformin and Eriomin^®^ (200 or 500 mg) and the control, showing that this combination influences the composition of the intestinal microbiota. Meng et al. [12] showed, in an animal model, alteration of the beta diversity with an intervention of eriocitrin (100 mg·kg^−1^·d^−1^), while we observed the same behavior with Eriomin^®^ associated with metformin.

The *Coriobacteriaceae* family increased in all treatments except for metformin plus 500 mg of Eriomin^®^ compared to the control. Some members of the *Coriobacteriaceae* family carry out conversions of bile salts, steroids, and polyphenols and may be beneficial for the host glucose metabolism [20]. A preclinical study has shown that the prevalence of 16S rRNA gene sequences assigned to the *Coriobacteriaceae* is dependent on host genotype and correlates positively with hepatic triglyceride levels or with plasma non-HDL cholesterol levels [21]. Furthermore, Liu et al. [22] observed a positive correlation between *Coriobacteriaceae* and the regulation of glucose metabolism after Roux-en-Y gastric bypass in patients with type 2 diabetes.

At the genus level, there was an increase in *Lachnoclostridium* in all treatments, except metformin plus 500 mg of Eriomin^®^ in relation to the control. Tsay et al. [23] observed, in a clinical study with diabetic individuals, a positive correlation between *Lachnoclostridium* ssp. and glycemic reduction.

Interestingly, the combination of metformin plus 500 mg of Eriomin^®^ increased *Bacteroides* and *Subdoligranulum* genera when compared to the control. The genus *Bacteroides* is a potential colonizer of the colon and represents a larger fraction of the intestinal microbiota [24]. In the diabetic mice model, the treatment with *Bacteroides acidifaciens* [25] and *Bacteroides uniformis* [26] improved glucose intolerance and insulin resistance. Together, these studies indicate that *Bacteroides* play a beneficial role in glucose metabolism in humans and experimental animals. In addition, *Bacteroides* species are SCFA producers, especially acetic and propionic acids [27].

Other studies have associated T2D with a decrease in butyrate producers, such as *Subdoligranulum* [28]. On the contrary, in more than a hundred overweight and obese individuals, *Subdoligranulum* was positively correlated with HDL-cholesterol and negatively with glycated hemoglobin (HbA1c). In the same study, the impact of just one cultured strain of *Subdoligranulum*, *S. variable* DSM 15176, was evaluated as a proof-of-concept with obese and diabetic mice, and, surprisingly, no significant difference was found in any of the markers of obesity or diabetes measured [29]. Interestingly, these results open several hypotheses, such as if the biological correlations of the genus *Subdoligranulum* refer to one or more specific strains, which have not yet been identified and cultivated, or if these correlations are just causality. Thus, the debate about the role of the genus *Subdoligranulum* in the development of T2D is not exhausted.

Despite the limited efficiency of metformin in reducing hyperglycemia, approximately 20–40%, there are numerous reports of undesirable side effects related to the digestive system, which include nausea, diarrhea, vomiting, abdominal discomfort, and loss of appetite [3]. Conversely, several papers have highlighted the influence of metformin on gut microbiota, with an increase in butyrate producers, such as the *Bifidobacterium* genus [30,31,32]. Our results showed an increase in *Bifidobacterium* and *Collinsella* genera and butyrate production after treatment with metformin.

Finally, we demonstrated a positive effect of Eriomin^®^ isolated or in association with metformin on the gut microbiota of pre-diabetic volunteers. These findings agree with previous studies realized by our research group [5,6,33] that revealed the efficacy of the citrus flavonoid eriocitrin in reversing hyperglycemia in patients treated with the nutraceutical Eriomin^®^, as well as a significant reduction of blood glucose associated with an increased in glucagon-like peptide-1 (GLP-1) and a decrease in inflammatory biomarkers.

## 4. Materials and Methods

### 4.1. Simulated Digestion in the Dynamic Microbiome Model

The Human Intestinal Microbial Ecosystem Simulator (SHIME^®^) is a simulator controlled by a computer [34]. For this experiment, SHIME^®^ was adapted to simulate a triplicate of the ascending colon, where the transverse and descending colon were replaced according to Salgaço et al. [35]. The pH, volumetric capacity, temperature (37 °C), and retention time were automatically controlled [36]. Throughout the system, nitrogen was added to obtain an anaerobic environment, and the pH value was corrected in each vessel using hydrochloric acid or sodium hydroxide to be in the range of 5.6–5.9 [37]. The compartments were colonized with fecal inoculum from the feces of four pre-diabetic adults (two male and two female). The inclusion criteria were subjects 30 plus years old, with a diagnosis of pre-diabetes, defined by fasting glucose between 6.0 and 6.9 mmol/L (100–125 mg/dL). The exclusion factors were the use of medications (antibiotics, antiglycemic, antihypertensive, etc.) or probiotics and/or prebiotics or having been ill in the last 30 days. A stool was prepared according to the previous description by Carvalho et al. [38].

### 4.2. Experimental Protocol

The total experimental period in the SHIME^®^ reactor lasted six weeks. For the microbiota stabilization period, the feed medium (300 mL) was inserted into the system daily and left to stabilize for 14 days [39]. After these two weeks, for one week, 240 mL of feeding medium plus 60 mL of pancreatic juice was inserted into the system daily (control). Subsequently, the experimental periods were divided into five treatments, performed in biological triplicate, as follows:

1. ERM200: Microbiota treated with Eriomin^®^ (200 mg/d) for seven days;
2. ERM500: Microbiota treated with Eriomin^®^ (500 mg/d) for seven days;
3. MTF: Microbiota treated with metformin (500 mg/d) for seven days;
4. MTF + ERM200: Microbiota treated with metformin (500 mg/d) + Eriomin^®^ (200 mg/d) for seven days;
5. MTF + ERM500: Microbiota treated with metformin (500 mg/d) + Eriomin^®^ (500 mg/d) for seven days.

### 4.3. Microbial Metabolites Analysis

To analyze SCFAs (acetic, propionic and butyric acids), 2 mL of colonic fermented samples are centrifuged (14,000 rpm for 5 min), and 1 mL of the supernatant stored for fatty acid analysis. The supernatants are diluted 1:1 with MilliQ water, filtered in Millex^®^ (0.45 μm), and then injected into an Agilent gas chromatograph (model HP-6890, Santa Clara, CA, USA) equipped with an Agilent selective mass detector (model HP-5975) using a DB-WAX capillary column (60 m × 0.25 mm × 0.25 μm) under the following conditions: temperature of injector = 220 °C, column = 35°C, 2 °C/min, 38 °C; 10 °C/min, 75 °C; 35 °C/min, 120 °C (1 min); 10 °C/min, 170 °C (2 min); 40 °C/min, 170 °C (2 min) and detector = 250 °C. Helium is used as a drag gas at a flow rate of 1 mL/min. Analytical curves are constructed using the stock solution of the acids of interest: acetic, propionic, and butyric acids. The samples are analyzed in triplicate per ascending colon replica before and after treatment. Data are expressed in mmol/g [40].

### 4.4. Microbiological Analysis Employing 16S rRNA Gene Sequencing

The extraction of bacterial DNA was performed using a DNeasy^®^ PowerSoil^®^ Pro Kit (QIAGEN, Hilden, Germany) following the manufacturer’s instructions. The DNA samples were then immediately frozen at −20 °C until molecular analysis. The library was prepared using primers for the V3–V4 region of 16S rRNA (~470 bp, amplified with primers 341F × 806R), and bacteria amplicons were sequenced using the Illumina platform (Novaseq6000 PE 250). The primers sequences used were 341F 5′-CCTAYGGGRBGCASCAG-3′ and 806R 5′-GGACTACNNGGGTATCTAAT-3′ [41].

Sequence data were processed and analyzed with QIIME (Quantitative Insights Into Microbial Ecology, version 2022.2.0 (https://qiime2.org/), accessed on 17 January 2023). On average, a total of 181,443 raw reads per sample were sequenced. Initially, during the demultiplex and trimming steps, the low-quality readings were removed, such as reads up to Q30 and reads with unsatisfactory length and chimeras were also removed with QIIME [42]. After this process, a data set containing an average of 27,154 raw reads per sample remained. The clean reads were used in the definition of the ASV (amplicon sequence variant). To measure the taxa present in the samples, a predictor model of the V3 and V4 regions was used (SILVA 138.99% OTUs from 515F/806R region of sequences). The operational taxonomic units (OTUs) were grouped by cluster readings with 99% similarity. The taxonomy was assigned to OTUs using the SILVA 138 reference database (https://www.arb-silva.de/, accessed on 17 January 2023). Heatmaps and bar plots of the relative abundance of the OTUs were generated with Python (version 3.7) through codes developed by the company ByMyCell Inova Simples Ltda (Ribeirao Preto, SP, Brazil).

The rarefaction curve was made using QIIME. The alpha diversity was calculated by applying different metrics. The Shannon, Simpson, and Fisher indices representing the species diversity were calculated. The Chao1 and ACE indices representing species richness were calculated. To evaluate the similarity of microbiota from different groups, the beta diversity was reported using weighted and unweighted UniFrac distances. The permutational multivariate analysis of variance (PERMANOVA) test was employed, using the Adonis test, to evaluate the differences in the beta diversity groups. Predictive functional profiling of the bacteria communities was identified by Phylogenetic Investigation of Communities by Reconstruction of Unobserved States 2 (PICRUSt2, version 2.4.2). The bacterial OTUs exported from QIIME2 in standard format were imported into PICRUSt2. Exploration analysis of the genomic data was carried out in Python (version 3.7) [43].

### 4.5. Statistical Analysis

Data are presented as the mean ± standard deviation (SD). Paired *t*-tests, one-way analyses of variance (ANOVAs), and Tukey’s post-hoc tests were used to analyze the results, with *p* < 0.05 considered statistically significant. Statistical analysis was performed using GraphPad Prism software, version 8.0 (La Jolla, CA, USA). 16S rRNA gene sequence analyses were performed in RStudio, version 3.2.4 [44], using the phyloseq package [45] to import sample data and calculate the alpha and beta diversity metrics. The significance of the categorical variables was determined using the non-parametric Wilcoxon test for two-category comparisons or the Kruskal-Wally’s test when comparing three or more categories. Principal coordinate plots were based on the PERMANOVA test to estimate *p*-values [46]. The *p*-values were adjusted for multiple comparisons using the FDR algorithm [47,48].

## 5. Conclusions

Eriocitrin, in combination with metformin, showed beneficial modulation of the intestinal microbiota of pre-diabetic volunteers. These results are promising for the use of the nutraceutical by pre-diabetics who are being treated with metformin since the modulation of the microbiota acts as one of the main actors in the prevention of the development of diabetes. However, clinical studies with the affected population are needed to confirm the findings of this preclinical study.
